# Metabolic Regulation of Cardiac Regeneration

**DOI:** 10.3389/fcvm.2022.933060

**Published:** 2022-07-08

**Authors:** Xuewen Duan, Xingguang Liu, Zhenzhen Zhan

**Affiliations:** ^1^Key Laboratory of Arrhythmias of the Ministry of Education of China, Institute of Heart Failure, Shanghai East Hospital, Tongji University School of Medicine, Shanghai, China; ^2^Department of Pathogen Biology, Naval Medical University, Shanghai, China

**Keywords:** heart regeneration, cardiomyocyte proliferation, fatty acid metabolism, glucose metabolism, amino acid metabolism, metabolism regulation

## Abstract

The mortality due to heart diseases remains highest in the world every year, with ischemic cardiomyopathy being the prime cause. The irreversible loss of cardiomyocytes following myocardial injury leads to compromised contractility of the remaining myocardium, adverse cardiac remodeling, and ultimately heart failure. The hearts of adult mammals can hardly regenerate after cardiac injury since adult cardiomyocytes exit the cell cycle. Nonetheless, the hearts of early neonatal mammals possess a stronger capacity for regeneration. To improve the prognosis of patients with heart failure and to find the effective therapeutic strategies for it, it is essential to promote endogenous regeneration of adult mammalian cardiomyocytes. Mitochondrial metabolism maintains normal physiological functions of the heart and compensates for heart failure. In recent decades, the focus is on the changes in myocardial energy metabolism, including glucose, fatty acid, and amino acid metabolism, in cardiac physiological and pathological states. In addition to being a source of energy, metabolites are becoming key regulators of gene expression and epigenetic patterns, which may affect heart regeneration. However, the myocardial energy metabolism during heart regeneration is majorly unknown. This review focuses on the role of energy metabolism in cardiac regeneration, intending to shed light on the strategies for manipulating heart regeneration and promoting heart repair after cardiac injury.

## Introduction

Heart failure is a burgeoning public health problem (1). It is a common and complex disease and has a poor prognosis in most patients. Ischemia is one of the main factors that contribute to heart failure. Following myocardial infarction (MI), the function and morphology of the cardiac cells begin to change. In the early phase of ischemia, cardiomyocytes lack oxygen and nutrients, resulting in a large-scale loss. Myofibroblasts in the heart became activated and produce collagen and other extracellular matrix components to compensate for the maintenance of the basic heart structure. In the later phase of ischemia, a large number of myofibroblasts accumulate, which thickens the ventricular wall and changes the mechanics of the heart, thereby impairing the cardiac pump function (2). As the proliferative capacity of adult cardiomyocytes is very low, they fail to repair the damaged area. This results in maladaptive cardiac remodeling and fibrosis, which ultimately progresses to an irreversible late-stage heart failure (3). With the exception of heart transplantation, current treatments, such as ventricular assist devices, fail to replenish the massive loss of cardiomyocytes after injury. Xenotransplantation and cardiac tissue engineering are achieving the encouraging progress for replacing damaged heart tissues (4). However, cardiac regeneration through altering cardiomyocyte fate plasticity is emerging as a promising approach to compensate for the loss of functional cardiomyocytes and repair cardiac functions.

A previous study revealed that in several non-mammalian lower vertebrates, endogenous cardiac regeneration occurs after cardiac damage (5). However, there has been a long-standing assumption that the mammalian heart is a terminally differentiated organ and that the adult mammalian heart cells are incapable of cell division and proliferation. Intriguingly, a study by Engel et al. (6) for the first time showed that adult mammalian ventricular cardiomyocytes could be induced not only to enter the cell cycle but also to undergo cytokinesis resulting in cell division. However, previous research showed that neonatal mice possessed remarkable endogenous cardiomyocyte proliferation and cardiac regeneration capacity before postnatal day 7 (P7) (7). Further evidence revealed that the heart of P3 mice/rat could not regenerate anymore, which might be due to cell cycle withdrawal and changes in energy metabolism occurring around P3 in mice and rats (8). Thus, establishing a neonatal mouse cardiac regeneration model provides a way to elucidate the mechanisms of cardiac regeneration. After birth, there is a shift in the source of nutrition from placenta-dependent to postnatal nutrition. This in turn triggers a dramatic change in the availability of energy substrates and leads to a shift in the metabolism of cardiomyocytes. Moreover, from embryonic to adult life, the oxygen status alters, thereby shifting the energy metabolism of cardiomyocytes. Interestingly, the shift in myocardial energy metabolism coincides with the time point of cardiomyocyte cycle withdrawal and loss of myocardial regenerative capacity in postnatal mice (9). This raises a question whether changes in metabolism are the cause of cell cycle exit or a consequence thereof. To sum up, these researches insinuate that myocardial metabolism plays a key role in the regenerative capacity of the heart.

In this review, we summarize the changes in major energy metabolic pathways, including fatty acid oxidation, glycolysis, amino acid metabolism, and tricarboxylic acid cycle (TCA), occurring during heart regeneration. We also discuss the key metabolic targets that point the way to research on cardiac regeneration.

## Energy Metabolism Is Involved in Heart Regeneration

### Oxygen Content Is Closely Related to the Regenerative Capacity in Lower Vertebrates

Lower vertebrates, such as zebrafish (*Danio rerio*) and axolotl, have the incredible potential for heart regeneration. The Mexican cavefish have been reported to regenerate their hearts after an injury (10). Zebrafish have the ability to regenerate cardiac muscles throughout their lifetime (11). After removing 20% of the ventricular volume, the heart can be completely regenerated within 2 months, with myocardial contractility returning to normal levels (11). The reasons for the high regenerative capacity of lower vertebrates might be divided into three aspects. First, the circulatory system of zebrafish is relatively hypoxic. This is because the zebrafish heart consists of a single atrium and a single ventricle, causing mixing of arterial and venous blood, which leads to low oxygen content in circulating blood (12, 13). Second, previous research found that centrosomes were intact in cardiomyocytes of zebrafish and salamander, while in mammalian cardiomyocytes they were disintegrated, impairing proper cell division (14). Finally, zebrafish cardiomyocytes are small and mononuclear throughout their life cycle and maintain proliferative potential (13, 15, 16). In addition, the oxygen content in the natural aquatic habitat of zebrafish is 1/30 of that in atmospheric air (17, 18). A previous study found that hypoxia promoted myocardial regeneration by altering the metabolic state. In contrast, exposing zebrafish to hyperoxic water at 45 kPa (vs. normoxic water at 21 kPa) inhibited myocardial regeneration (19). In addition, the change in environmental oxygen level from embryonic to adult stage triggers a switch in energy metabolism of the postnatal heart, which generates mitochondrial reactive oxygen species (ROS). The DNA damage response (DDR) is triggered, and the cell cycle of postnatal cardiomyocytes is thus stalled (17). Moreover, a research found that ROS altered the activity of metabolically critical enzymes and hence played a role in cardiac regeneration of zebrafish. Since elevated H_2_O_2_ levels are detrimental, investigators identify protein tyrosine phosphatase 1b (Ptp1b) as a downstream target of ROS and propose that inhibition of Ptp1b promotes myocardial regeneration (20). In addition, MSI-1436, a small molecule inhibitor, selectively represses Ptp1b and hence promotes regeneration (21). Dual-specificity phosphatase 6 (Dusp6), a member of the PTP family, is sensitive to the redox effects of ROS. Moreover, the change in Dusp6 expression correlates with the regenerative window of the heart, which suggests Dusp6 might have a key role in the early cardiac regeneration process (22). Furthermore, by single-cell transcriptome analysis of regenerated zebrafish myocardium, researchers found that cardiomyocytes in the proliferative border zone underwent a shift in energy metabolism after heart cryoinjury (23). This suggests that proliferation and regeneration of cardiomyocytes after cardiac injury in zebrafish involve energy metabolism.

### Energy Metabolism in Cardiac Regeneration of Mammals

Morphology of murine neonatal heart is similar to that of zebrafish heart (24). Hence, researchers turned their attention to find out whether mammalian heart could regenerate after damage. The size of human cardiomyocyte increases approximately 8.6-fold during the first 20 years of life (25). Although adult cardiomyocytes can temporarily self-renew, their self-renewal rate is extremely low (26). In addition, the regeneration ability of adult cardiomyocytes is very limited and is not enough to repair the damaged myocardial tissue. Hence, adult mammalian myocardial tissue cannot regenerate after heart damage. However, recent data suggest that cardiomyocyte renewal occurs in adulthood in mammals including humans (27, 28). Interestingly, in 2011, researchers discovered that neonatal mice had an amazing myocardial regenerative potential (7). This study showed that 1-day-old newborn mice heart had the ability to regenerate resected myocardium after apical resection. Nonetheless, this regenerative ability is lost after 7 days of birth (7).

Further, on investigating the reasons for reduced regenerative capacity of adult mice, researchers found that differences in the type of energy metabolism were influential factors that determined the ability of the myocardium to regenerate. There are two main ways by which cardiac energy in mice is altered. On the one hand, a change in oxygen status from embryonic to postnatal causes a shift in cardiac metabolism. Due to exposure to relatively hypoxic environment, embryonic blood shunt circulation in the mammalian heart results in significant mixing of arteriovenous blood (29). However, after birth, the shift in circulation and the rapid increase in arterial oxygen change the oxygenation status of cardiomyocytes within minutes (17). Therefore, mammalian cardiomyocytes are capable of generating energy through metabolic conversion to adapt to high energy demands after birth (30). The main source of adenosine triphosphate (ATP) is cardiac metabolism, and ATP maintains cardiac homeostasis and function (31, 32). Mitochondria perform energy conversion, and more than 95% of the ATP is produced through energy substrates for the heart (33). After birth of mice, energy metabolism of neonatal cardiomyocytes changes, and the number of cardiomyocyte mitochondria dramatically increases in the adult heart (34). One research found that mitochondrial DNA copy number was lower in the neonatal murine heart compared with the adult murine heart (17). At present, many studies focus on understanding the regulation of mitochondrial metabolism in postnatal cardiomyocyte cycle arrest and influencing future regeneration strategies.

**FIGURE 1:**
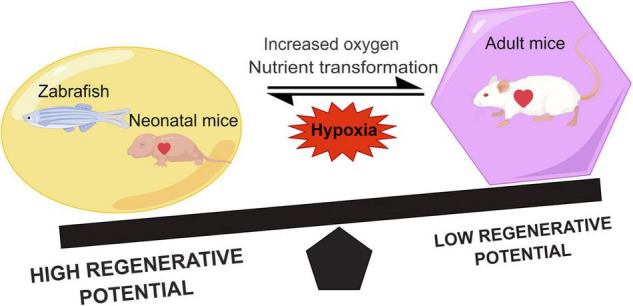
Comparison of the regenerative capacity of representative lower vertebrates, neonatal, and adult mammals. Hypoxia can promote heart regeneration and increase the regenerative capacity. During the growth of neonatal mice, the shift in metabolic state of the heart due to the increased oxygen and nutrients leads to a decrease in the myocardial regenerative capacity.

On the other hand, nutrient supply is altered after birth of mice (35), and the shift in metabolic pattern causes cardiomyocyte maturation and cell cycle exit (36). Previous studies on porcine and rabbit hearts indicated that postnatal cardiomyocytes underwent a shift in energy source compared with embryonic cardiomyocytes due to different nutrients (37, 38). Therefore, the increased oxygen content at sharp atmosphere stress and changes in metabolic matrix postnatally together alter the type of energy metabolism in cardiomyocytes and reduce their proliferation capacity (Figure 1). In all, the regenerative capacity of the myocardium is related to the metabolism of cardiomyocytes.

## Hypoxia Regulates Metabolic Reprograming to Promote Cardiac Regeneration

Hypoxia refers to reduced and insufficient oxygen supply, and it influences myocardial energy metabolism and cardiac contractile function (39, 40). Chronic hypoxia decreases the activity of fatty acid oxidase (40), which in turn reduces fatty acid uptake and oxidation in mitochondria (41). In addition, it increases glycolysis and glycolytic enzyme activity (42). Moreover, chronic hypoxia decreases mitochondrial utilization of fatty acid and pyruvate substrates and reduces the enzymatic activities of electron transport chain (ETC) complexes I, II, and IV in the mitochondria of cardiomyocytes, thereby lowering the production of ROS (43). Hence, the regenerated cardiomyocytes show characteristics of proliferative cardiomyocytes in the embryonic stage, which exhibits hypoxic environment.

Professor Sadek’s team found that moderate hypoxia promoted myocardial regeneration after myocardial infarction (44). They reported that gradually reducing inhaled oxygen by 1% and maintaining oxygen concentration level at 7% for 2 weeks, reduced ROS production and DDR, however, restarted mitosis in cardiomyocytes. Hypoxia-inducible factor (HIF)-1 family adjusts the cells to hypoxic environment. HIF-1 is a class of specialized heterodimers composed of unstable alpha subunits (HIF-α). HIF-1α protein is steady under hypoxic conditions and activates transcription of multiple genes involved in glycolysis, fatty acid metabolism, mitochondrial metabolism, and cell cycle regulation (45). Stem cells or progenitor cells of some organs are relatively hypoxic and maintain their normal physiological functions by stabilizing the HIF-1α subunit (46). In 2012, researchers found that hypoxia promoted cardiomyocyte dedifferentiation and myocardial regeneration in adult zebrafish, and HIF-1α played the important role in this (19). However, another study found that the complete activation of HIF-1α signal led to the dilated cardiomyopathy and heart failure (47, 48). These findings suggest that the moderate activation of HIF-1α hypoxia signal may be a potential strategy for heart regeneration. In addition, the authors found that HIF-1α deficiency impaired glycolysis and inhibited proliferation of hypoxic fetal cardiomyocytes, which in turn led to transient reprograming of amino acid metabolism and activation of HIF2 (19, 49, 50). This shows that the embryonic heart is metabolically flexible.

In conclusion, chronic severe hypoxia enhances the expression of glycolytic and cell cycle genes, whereas it inhibits the expression of fatty acid oxidation genes and cell cycle inhibitory genes (51), and thus, it metabolically reprograms embryonic heart. These genetic and metabolic changes promote myocardial regeneration, which is referred to as the hypoxia-induced cardiomyocyte proliferation and the infarcted zone revascularization.

## Different Types of Energy Metabolism Affect the Myocardial Regenerative Capacity

**FIGURE 2:**
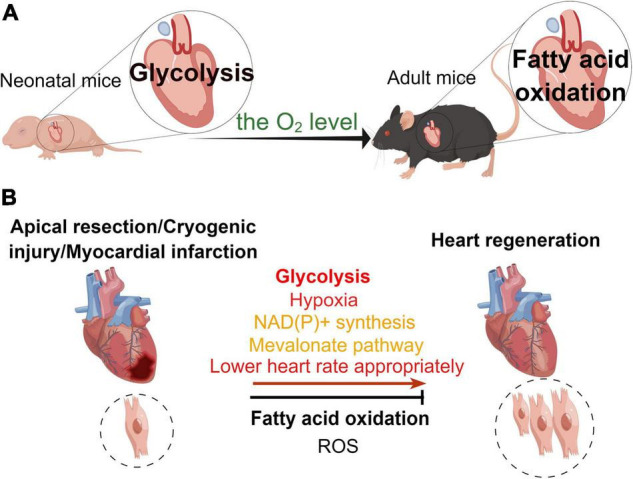
Different metabolic patterns in physiological and pathological states affect the myocardial regenerative capacity. **(A)** Under physiological conditions, the main mode of metabolism in the murine neonatal heart is glycolysis, whereas that in the hearts of adult mice is fatty acid oxidation. **(B)** In the pathological state, glycolysis, hypoxia, NAD(P)+ synthesis, mevalonate pathway, and appropriate reduction in heart rate promote heart regeneration, while fatty acid oxidation and reactive oxygen species (ROS) inhibit myocardial regeneration in the neonatal mouse heart regeneration models (apical resection, cryoinjury, or myocardial infarction).

The heart is the most energy-intensive organ of the body (52). In the physiological state, the heart of adult mouse produces ATP in two ways to maintain its contractile function. Mitochondrial oxidative metabolism produces approximately 95% of the cardiac energy, while glycolysis produces only 5% of it. About 40–60% of the energy in mitochondrial oxidative metabolism is generated through free fatty acids and glucose metabolism, while the remainder of this is produced by the oxidation of ketones, lactate, and amino acids (53, 54). Compared with glucose oxidation, fatty acid oxidation requires more oxygen. Hence, the aerobic oxidation efficiency of glucose is higher than that of fatty acids (53). During the perinatal period, glycolysis and lactate oxidation are the main sources of cardiac ATP (55–57), while fatty acid oxidation produces only a small fraction of ATP (55). In the early postnatal period (from P1 to P7), glycolysis produces almost half of the ATP for the neonatal murine heart (55). While 7 days post-birth, glycolysis gradually decreases, providing only a small amount (10%) of ATP for cardiomyocyte metabolism. However, there is a significant increase in fatty acid oxidation to maintain cardiomyocyte energy metabolism (38, 55). In the neonatal period, the source of cardiac energy is β-oxidation of fatty acids, which produces ATP at levels close to those found in the heart of adult animals (58). The researchers measured metabolite levels, such as fatty acids, in the cardiac arteries of fetal, newborn (1–4 days), and juvenile (7 weeks) lambs (57, 59). They found that fatty acid flux was zero in the embryonic heart. Interestingly, there was an increase in fatty acids in the myocardium of newborn lambs, but there was no net flux of fatty acid (57). And the flux of fatty acids was increased in the juvenile lambs (57). Glucose and lactate were sources of energy metabolism for embryonic and neonatal (3–15 days) lamb hearts (60). In addition, a research found that fatty acids promoted proliferation of P4 cardiomyocytes but inhibited proliferation of P5 cardiomyocytes (61). In all, different developmental stages and oxygen levels result in altered energy sources for the mouse heart. This leads to varying metabolic states of cardiomyocytes that affect their proliferative capacity (Figure 2).

### Fatty Acid Metabolism and Cardiac Regeneration

Studies indicate that fatty acid oxidation is the main source of cardiac energy for the adult heart (62). After entering cardiomyocytes, fatty acids generate ATP mainly through oxidation *via* the TCA in the mitochondria. Among all energy substrates, fatty acids produce the highest ATP content and have the highest oxygen demand. Therefore, fatty acids are myocardial energy substrates with the lowest metabolic efficiency (ATP production/oxygen consumption) (33).

A research found that feeding fatty acid-deficient milk to postnatal mice prolonged the proliferation window of their hearts (63). Carnitine palmitoyltransferase 1 (CPT1) transfers fatty acids from the cytoplasm to the mitochondria and is the key enzyme regulating fatty acid oxidation. CPT1 activity in the neonatal murine heart is very low, while it increases significantly in the heart of a 7-day-old mouse, which coincides with the time when mammalian cardiomyocytes lose their ability to proliferate (64). One research found that etomoxir (ETO) could inhibit the activity of CPT1, thereby reducing fatty acid oxidation and promoting proliferation of neonatal mouse cardiomyocytes and heart regeneration in neonatal mice (61). Previous study showed that the low expression of carnitine palmitoyltransferase 2 (CPT2), which is an essential enzyme for fatty acid oxidation (65), could promote cardiomyocyte proliferation in P14 mice (66). Acyl-CoA synthetase long-chain family member 1 (ACSL1) mediates the uptake of cellular lipids and is a key enzyme regulating lipid metabolism. On examining the expression of glycolipid-related enzymes in heart tissue of mice at different ages, it was found that ASCL1 expression increases with the age of mice (67). Inhibition of ACSL1 expression with a corresponding decrease in cardiomyocyte lipid uptake in cardiomyocytes of adult mice post-MI and primary neonatal mouse cardiomyocytes upregulated the expression of cell cycle-activating genes, but downregulated the expression of cell cycle-inhibiting genes (67).

However, the view that fatty acid oxidation inhibits myocardial regeneration is still debatable. A research found that peroxisome proliferator-activated receptor alpha (PPARα)-mediated β-oxidation of fatty acid promoted primary neonatal mouse cardiomyocyte hypertrophy and maturation and enhanced the presence of binucleated cardiomyocytes at postnatal day 5. Moreover, it caused the withdrawal of cardiomyocytes from cell cycle (61). Nevertheless, it is interesting to note that PPARα-mediated β-oxidation of fatty acid promoted G0/G1 cell cycle entry rate and proliferation of cardiomyocytes in mice at postnatal day 4 (61). What makes fatty acid oxidation has opposite regulatory effects on myocardial regeneration? In this context, the authors speculate that the reduced proliferation rate of cardiomyocytes at postnatal day 5 is due to the presence of binucleated cardiomyocytes and cardiomyocytes leaving the cell cycle (61). These results suggest that the effect of fatty acids on myocardial regeneration is inconclusive and needs to be studied in the future.

### Glucose Metabolism and Cardiac Regeneration

Glycolysis is the primary mode of energy metabolism in the neonatal murine heart. A previous study reported that glycolysis promoted myocardial regeneration in adult zebrafish (68). Glucose is an important fuel and produces ATP through cytoplasmic glycolysis and mitochondrial oxidation in the heart. In the heart, glucose transporter 1 (GLUT1) and GLUT4 transport glucose into cardiomyocytes. Under physiological conditions, GLUT1, whose the expression increases by HIF-1α (69), is the main glucose transporter in embryonic and neonatal hearts (70, 71). A research found that overexpression of GLUT1 promoted glycolysis in the neonatal murine heart (72, 73) and increased nucleotide synthesis, thereby promoting myocardial regeneration by enhancing glucose metabolism after cryogenic injury in neonatal mice at P21 and P40 (9).

The key glycolytic enzymes play important roles in myocardial regeneration. Phosphofructokinase 2 (PFK2) regulates the level of fructose-2,6-bisphosphate (Fru-2,6-P2) by phosphorylating fructose-6-phosphate (F6P) during glycolysis. A research revealed that PFK2 could increase the contractility of hypoxic cardiomyocytes in mice, suggesting that it affects the metabolism of cardiomyocytes while maintaining their function (74). Pyruvate dehydrogenase kinase (PDK) inhibits the activity of pyruvate dehydrogenase (PDH). Previous studies found a significantly high expression of PDK in the infarct area of the adult heart (71) and zebrafish (68) after cryogenic injury of the heart. Moreover, PDK4 deletion promoted cardiac regeneration in adult mice after MI and proliferation of primary adult mouse cardiomyocytes by increasing glycolysis (63). In contrast, a report implied that PDK3 overexpression promoted cardiomyocyte proliferation by enhancing glycolysis during the early stages of cardiac regeneration in zebrafish, while there was no scar repair in the later stages (68). What is the reason for the difference between early and late stages? Since PDK3 overexpression can promote cardiomyocyte proliferation, can it also promote maturation of new cardiomyocytes to replace injured cardiomyocytes, and thus promote scar repair? Of note, PDK3 and PDK4 are both isozymes of PDK, so why do they exhibit opposite effects on cardiomyocyte proliferation? Their distributions are different, but the mechanisms involved require further study. Notably, studies have shown that M2-pyruvate kinase (PKM2) can promote the phosphopeptide pathway and inhibit oxidative stress (75, 76). A research found that knockout of PKM2 in mice post-MI promoted catenin beta 1 (Ctnnb1) translocation to the nucleus and trans-activation of key genes that mediate cell cycle progression (77). In zebrafish, the *Pkma2* gene encodes M2-pyruvate kinase. It has been found that pkma2 knockdown in zebrafish decreases glycolysis and thereby inhibits cardiomyocyte proliferation (68). Magadan’s team found that overexpression of PKM2 promoted cardiomyocyte proliferation and regeneration after MI in adult mice, by increasing the expression of glucose-6-phosphate dehydrogenase (G6PD) (78). Therefore, PDK and PKM2 serve as promising treatment strategies for promoting cardiac repair and regeneration.

Interestingly, some key regulatory genes can regulate cardiac regeneration by promoting glycolysis. Myeloid ecotropic viral integration site 1 (MEIS1) is an important transcription factor that regulates the cardiomyocyte cycle. A research found that myocardium-specific knockout of MEIS1 in neonatal mice could extend the proliferation window of cardiomyocytes and that these cardiomyocytes could re-enter the cell cycle (79). Conversely, another finding indicated that inhibiting MEIS1 expression in primary fetal and neonatal sheep cardiomyocytes resulted in the increased mitochondrial activity and decreased glycolytic genes, leading to the enhanced cardiomyocyte maturation (80). The discrepancy in MEIS1 function between mice and sheep may be related to the differences in cardiomyocytes themselves from these two species. Further studies are needed to understand the mechanism of MEIS1 regulation on the metabolic state as well as the cell cycle of cardiomyocytes.

It is worth noting that cardiomyocyte proliferation-related pathways can also affect glycolysis. Furthermore, Yes-associated protein (YAP), a component of the Hippo pathway, is involved in glycolytic metabolism during heart development (81). A previous study found that the defect in Hippo pathway could activate Yap signaling to promote cardiomyocyte proliferation in adult mice (82–84). Studies on adult cardiomyocytes showed that direct and indirect target genes of activated Yap were associated with glycolysis and mitochondrial metabolism (84–86). In addition, the interaction between neuregulin 1 (NRG1) and its receptors, ErbB2 or ErbB4, facilitated glycolysis and inhibited mitochondrial oxidative phosphorylation (87), which was vital for cardiomyocyte proliferation in zebrafish (88) and newborn mice (89). Researchers showed that ErbB2 affected cardiac regeneration *via* downstream activation of YAP (81). Moreover, Wnt/β-catenin and ERK/MYC signaling pathways promoted glycolysis and cell proliferation by upregulating cyclin–CDK complex in neonatal mouse cardiomyocytes and immature human pluripotent stem cell-derived cardiomyocyte (hPSC-CM) (90, 91). Overexpression of c-Myc could promote glycolysis and cardiomyocyte proliferation in mice during both gestation and adult life (92). In contrast, Wnt/β-catenin signaling in adult mice was cardioprotective but did not induce cardiomyocyte proliferation (91). Thus, the role of Wnt/β-catenin signaling in mice at different stages depends on the metabolic state of cardiomyocytes (91). Taken together, these pathways and genes can regulate cardiomyocyte proliferation by influencing glycolysis. However, which gene or component of the glycolytic pathway is affected is not yet elucidated. Moreover, whether these pathways only affect glycolysis or also affect lipid and other metabolic pathways remains to be determined.

In all, these studies indicate that glycolysis plays an essential role in modulating cardiomyocyte proliferation and cardiac regeneration after injury. As a result, targeting glucose metabolism is an interesting strategy to promote adult heart regeneration.

### Amino Acid Metabolism Followed by Heart Regeneration

Cardiomyocytes require protein synthesis during growth, after birth, and during maturation. The increased protein synthesis results in the increased amino acid metabolism. Amino acid oxidation is also a source of cardiac ATP, with branched-chain amino acids (BCAAs) playing a major role. BCAA includes three amino acids, namely, leucine, isoleucine, and valine. Although BCAA oxidation produces only 2% of the total ATP generated by the heart (93), BCAAs play an important role in regulating cardiac signaling pathways, including insulin and mTOR signaling pathways (94).

Metabolomic analysis of mouse heart tissues at different developmental stages revealed that BCAAs showed differential levels in the heart at different developmental stages (95). Valine, leucine, and isoleucine concentrations gradually increased postnatally reaching a peak at day 9 and then decreased until they reached to the postnatal day 1 (P1) level on P23 (95). Most of the differentially expressed genes (DEGs) directly correlated with BCAA concentrations and were upregulated at the mRNA level between P9 and P23 (95). Therefore, we speculate whether inhibiting enzymes of the BCAA breakdown pathway could promote cardiomyocyte proliferation.

Glutamine is an amino acid transporter. Elevated glutamine expression in zebrafish and neonatal mouse cardiomyocytes activated the amino acid-driven mTOR signaling pathway, which promoted mitochondrial maturation and regulated cardiomyocyte proliferation (96). In addition, high concentrations of leucine and glutamate were found to activate the mTOR pathway and promote regeneration (96). Since leucine concentration rises in the postnatal week, does the concentration of BCAA also affect the ability of cardiomyocytes to proliferate? Researchers found that cardiomyocyte injury initiated Wnt/β-catenin signaling during zebrafish cardiomyocyte regeneration, which in turn initiated mTOR activation and metabolic remodeling (97). As previously described, Wnt/β-catenin signaling pathway promoted glycolysis and activated mTOR signaling pathway together with BCAAs (91, 98). This suggests that metabolic pathways are not independent but interact with each other. Thus, alterations in amino acid metabolism in cardiomyocytes affect their proliferation. However, studies in this research area are limited and need to be investigated in the future.

### Tricarboxylic Acid Cycle Metabolites and Cardiac Regeneration

Tricarboxylic acid cycle occurs in the mitochondrial matrix. It is the metabolic center of the carbohydrate–lipid–amino acid linkage and the last metabolic pathway of the three nutrients. The transformation of energy metabolism and an increase in mitochondrial DNA after birth of mice lead to a significant increase in ROS produced by mitochondria (17). This increased ROS production damages proteins, lipids, and DNA and leads to cell cycle arrest (99–102). Therefore, clarifying the role of mitochondrial metabolites in regulating metabolic switch is essential for identifying metabolic targets that will promote adult heart regeneration.

To begin with, the primary approach to promote cardiomyocyte proliferation by inhibiting mitochondrial metabolism is to suppress ROS production by mitochondrial oxidation. Studies have reported that overexpression of mitochondrial catalase (mCAT) and the ROS scavenger *N*-acetylcysteine (NAC) system reduces DDR and promotes cardiomyocyte proliferation (17). Martin’s team found that the activation of the antioxidant response after cardiac injury could reduce ROS and thereby promote cardiac repair (84). In cardiomyocytes, nuclear factor-erythroid-2-related factor 2 (Nrf2) directly regulates the expression and subcellular location of paired-like homeodomain 2 (Pitx2). Genome analysis indicated that Pitx2 activates genes in response to oxidative response by recruiting YAP (103). The knockdown of Pitx2 in the neonatal mouse heart leads to the failure of regeneration after cardiac injury. This suggests that antioxidant stress response pathways are critical for cardiomyocyte proliferation and cardiac regeneration (84). These include genes encoding ETC components and ROS eliminator that protect cells from ROS damage and promote cardiomyocyte regeneration (84, 103). Pei et al. (104) confirmed that hydrogen sulfide (H2S) could scavenge ROS, and hence, cardiomyocytes could re-enter the proliferation cycle. Researchers used propargylglycine (PAG) to inhibit H2S synthesis and found that it promoted the deposition of ROS in the murine neonatal heart and inhibited proliferation of cardiomyocytes (104). In contrast, sodium hydrosulfide hydrate (NaHS), an H2S donor, could reduce ROS accumulation and promote cardiomyocyte proliferation and regeneration by alleviating H2S-mediated cardiomyocyte injury (104). These findings reveal that antioxidant pathways play an important role in the regeneration of the heart. In addition, cardiac sarcomere contraction after birth promoted mitochondrial metabolism and activated p53 to produce ROS and DNA damage responses (105). It is known that p53 causes cardiomyocytes to exit the cell cycle by repressing *Cyclin B1* gene (106). We speculate that cardiac sarcomere contraction affects the proliferative capacity of cardiomyocytes by regulating their metabolism. However, this speculation remains to be validated. Overall, sarcomere disassembly in cardiomyocytes can preclude ROS production and promote proliferation of cardiomyocytes (105).

Succinate dehydrogenase (SDH) converts succinate to fumarate in the TCA cycle. During ischemia, succinate concentration increases and inhibits SDH activity. A previous study revealed that injecting succinic acid in neonatal mice inhibited the proliferation of cardiomyocytes. In contrast, malonate, an SDH inhibitor, prolonged the regeneration window of the heart after birth and promoted the proliferation of cardiomyocytes and heart regeneration in adult mice after MI (107). Similarly, malonate injection in neonatal mice promoted myocardial regeneration after an injury (108). In addition, malonic acid induced the formation of a new coronary vascular system within the infarcted tissue (108), given that glycolysis promoted angiogenesis through apical cell formation (109). In short, malonate promotes proliferation of cardiomyocytes and vascularization would be a candidate therapeutic target. In terms of clinical translation, delivery of SDH inhibitors directly to the heart may be more effective because SDH deficiency can lead to tumorigenesis (108). Nonetheless, the role of other critical enzymes of the TCA cycle is not yet clear. In short, these studies suggest that in addition to glycolipid metabolism, changes in the activity of critical enzymes of the TCA cycle also have different impacts on cardiomyocyte proliferation.

## Several Biosynthetic Pathways Affect the Myocardial Regenerative Capacity

Interestingly, researchers found that in addition to classical metabolic pathways, several biosynthetic pathways could affect cardiomyocyte proliferation. A study revealed that simvastatin inhibited cardiomyocyte proliferation by inhibiting the mevalonate pathway of isoprene synthesis in cardiomyocytes of human-induced pluripotent stem cell (hiPSC)-cardiac organoids (110). Previous studies found that the expression of genes that regulated ancillary biosynthetic pathway enzymes increased in cardiomyocytes in response to cell cycle stimulation by four cell cycle factors, namely, cyclin B1, cyclin D1, CDK1, and CDK4. These enzymes were related to the hexosamine biosynthesis pathway (HBP), serine biosynthesis pathway, protein *O*-GlcNAcylation, and those involved in NAD(P)+ (111). Researchers found that overexpression of *O*-GlcNAcase (OGA) was associated with HBP and prevented cardiomyocyte cycle entry and progression, while knockdown of nicotinamide phosphoribosyltransferase (NAMPT) inhibited cycle entry of human iPSC cardiomyocytes (hiPSC-CM) (111). In addition, investigators found that overexpression of phosphoenolpyruvate carboxykinase 2 (PCK2), a gluconeogenic pathway enzyme that catalyzed the transformation of oxaloacetate to phosphoenolpyruvate, promoted proliferation of hiPSC-CM (111). In general, the crossover point metabolites in these synthetic pathways are provided by the glycolytic pathway, which again highlights the importance of the glycolytic pathway in the process of myocardial regeneration.

Previous research found that cardiomyocyte-specific overexpression of PPARδ induced cell cycle progression in cardiomyocytes after MI in mice and improved cardiac function (112). In addition, carbacyclin, a PPARδ activator, induced proliferation of neonatal and adult rat mononuclear cardiomyocytes *via* (PPARδ)/PDK1/Akt/GSK3β/β-catenin pathway (112). Glycogen synthase kinase-3β (GSK-3β) is a serine/threonine kinase originally identified as a key enzyme in glycogen synthesis. The authors do not indicate whether myocardial metabolism is altered during PPARδ-mediated myocardial regeneration. In addition, we cannot speculate whether PPARδ ultimately promotes myocardial regeneration by enhancing glycogen synthesis during this process. In contrast, previous studies have shown that PPARβ/δ overexpression in the heart increases glucose utilization by enhancing the expression of glucose transporter protein type 4 (Glut4) (113, 114). Can this be considered that PPARδ promotes cardiomyocytes to enter the cell cycle by enhancing glycolysis? This should be further delved into future studies.

In addition to its ability to regulate glycolysis, PKM2 has been reported to redirect glucose carbon flow to oxidative pentose phosphate pathway (PPP), thereby reducing oxidative stress and causing increased expression of cell cycle genes in postnatal cardiomyocytes (78). It has been shown that overexpression of the glucose transporter protein Glut1 leads to an increase in glucose metabolites and thus nucleotide supply in heart regeneration of neonatal mice (9). Recently, researchers have found that metabolic pathways can combine with biosynthetic pathways to affect the cardiomyocyte proliferation. Moderate heart rate reduction (HRR) can induce cardiomyocyte proliferation by altering the metabolic pattern of cardiomyocytes (115). On the one hand, glucose metabolism enzymes exert non-enzymatic activity to promote cell cycle progression. On the other hand, the PPP is activated to supply the biosynthetic metabolic demand required for promoting cardiomyocyte proliferation (115). In addition, researchers found that lowering the heart rate upregulated the expression of cyclin D1. Moreover, they also observed an increase in the nuclear transport of PKM2, which promoted cyclin D1 and thus restarted cardiomyocyte proliferation (115). This suggests that the three biological properties of cardiomyocytes, namely continuous rhythmic beating, unique energy metabolic pattern, and limited proliferative capacity, are intrinsically linked. In addition, PPP may be a prospective regenerative strategy for cardiac injury. Overall, reducing the heart rate of cardiomyocytes can affect their energy metabolic patterns and thus promote cardiac regeneration. However, some questions have not been explored yet. For example, what specific molecules and signaling pathways of PPP are affected by HRR regulation? Are there any other biosynthetic pathways that are also affected? Furthermore, this suggests that future studies combine metabolism and synthesis, rather than a single aspect, to explain the phenomenon of cardiac regeneration.

## Differences in Energy Metabolism Between the Fetal Heart and the Failing Heart

Heart failure is the end stage of many heart diseases, including the ischemic cardiomyopathy and non-ischemic cardiomyopathy. In this regard, MI is a common ischemic cardiomyopathy, while cardiac hypertrophy due to pressure-loaded cardiomyopathy is a common non-ischemic cardiomyopathy. These processes include a wide range of remodeling in metabolism, cardiac structure, and cardiac electrophysiology (116). There is growing evidence that metabolic remodeling precedes most pathological processes and may play an important role in myocardial hypertrophy and heart failure (33, 117–119). In the failing heart, mitochondrial ROS production increases and also mitochondrial autophagy decreases. This results in the impaired mitochondrial function and reduced mitochondrial oxidative metabolism (120, 121). Hence, to increase the ATP supply, there is a compensatory increase in glycolysis (122). In addition, the energy metabolism shifts to a “fetal”-like pattern of energy substrate metabolism after cardiac hypertrophy (123, 124). That is, glycolysis increases and fatty acid oxidation decreases in pressure-overload cardiac hypertrophy (122, 125).

Although major changes in glycolysis and fatty acid oxidation in both failing and hypertrophic hearts resemble the major metabolic pattern of the embryonic heart, the cardiomyocyte proliferation capacity varies. For this reason, the specific metabolic patterns in the embryonic and failing hearts are intrinsically different. The primary difference is the substrate of energy metabolism and the way of metabolism. In the ischemic heart, anaerobic glycolysis is the main source of cardiac ATP and leads to the production of lactate (126). In the absence of blood perfusion, glucose is not available to cardiomyocytes, and hence, previously stored glycogen is used to produce ATP (127). Since glycolysis produces less ATP than oxidative phosphorylation, ATP is consumed more than it is produced. The intracellular lactic acid accumulates, leading to cellular acidosis, and inhibits enzymes of the glycolytic pathway (116). Hence, the efficiency of glycolysis decreases during prolonged ischemia (127). Glycolysis ceases despite the availability of glycogen stores in cardiomyocytes (127). In addition, the lack of blood perfusion leads to the accumulation of intracellular protons that inhibits glycolysis in a feedback manner (128, 129). However, during the embryonic period, the heart obtains energy mainly through glucose. Due to low oxygen concentration during the embryonic period, the embryonic heart produces energy mainly through anaerobic glycolysis, which promotes heart regeneration (68). This suggests that the substrates of energy metabolism are different in the ischemic and regenerative heart.

On the one hand, researchers found that aerobic glycolysis was the main source of cardiac ATP in pressure-overload-induced heart failure (130–132), whereas, anaerobic glycolysis was the main source of cardiac ATP during myocardial regeneration in neonatal mice (133). On the other hand, glucose oxidation was lower in hypertrophied hearts than that in non-hypertrophied hearts (134, 135). When the heart rates were appropriately reduced, P1 cardiomyocytes were more dependent on glucose oxidation (115). This suggests that glucose oxidation promotes myocardial proliferation. Thus, regenerative and hypertrophic hearts are metabolized in different ways. Besides, researchers tested 13 key genes for energy metabolic substrates, including “adult” isoform genes and “fetal” isoform genes. They found that the expression of “adult” isoform of metabolic genes was reduced in the failing adult heart (124). This suggests that the reversion of the metabolic pattern in the failing heart is similar to the metabolic pattern of the embryonic heart (124). However, some studies had different conclusions. It was found that fatty acid oxidation (FAO) was either unchanged or increased, whereas glycolysis was either unchanged or decreased in the hypertrophied heart (132). Another research showed that myocardial FAO was unchanged, glycolysis was slightly reduced, and glucose and lactate oxidation was decreased in angiotensin II-induced myocardial hypertrophy (136). These discrepancies suggest that the metabolism of failing and regenerating hearts is different. Hence, the failing cardiac metabolism evolving from infarction and myocardial hypertrophy is different from the cardiac metabolism of neonatal mice.

## Concluding Remarks and Perspectives

**FIGURE 3:**
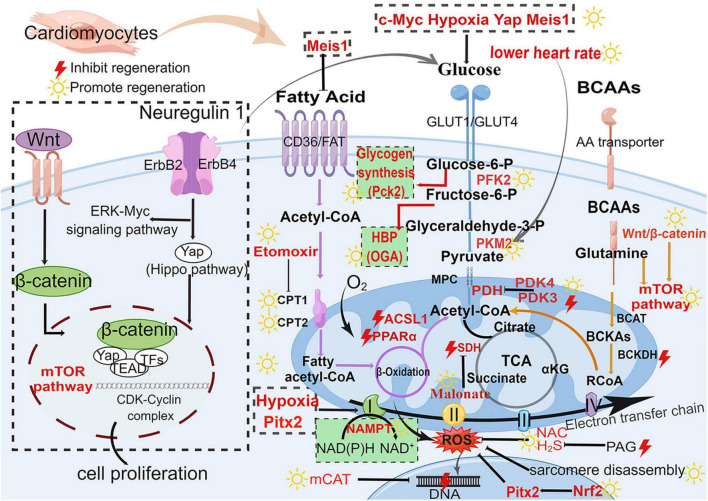
Major metabolic pathways and signaling pathways in heart regeneration after cardiac injury. Glucose metabolism (blue), fatty acid metabolism (purple), BCAA metabolism (orange), and biosynthetic pathways (green background). The boxed section shows the factors affecting the metabolic pathways. Wnt/β-catenin pathway and Nrg1-ErbB pathway affect the metabolism of heart regeneration. Acetyl-CoA is the final effector of these three metabolism pathways (fatty acid oxidation, glycolysis, and amino acid metabolism) and regulates the initiation of the TCA cycle. GLUT, glucose transporter type; Glucose-6-P, glucose-6-phosphate; Fructose-6-P, fructose-6-phosphate; Glyceraldehyde-3-P, glyceraldehyde-3-phosphate; PFK, phosphofructokinase; PKM2, M2-pyruvate kinase; PDK, pyruvate dehydrogenase kinase; PDH, pyruvate dehydrogenase; CD36, cluster of differentiation; FAT, fatty acid transport carrier; CPT, carnitine palmitoyltransferase; BCAAs, branched-chain amino acids; BCAT, branched-chain amino acid transferase; BCKA, branched-chain alpha-keto acids; BCKDH, branched-chain alpha-keto acid dehydrogenase; mTOR, mammalian target of rapamycin; TCA, tricarboxylic acid cycle; SDH, succinate dehydrogenase; αKG, α-ketoglutarate; ROS, reactive oxygen species; H_2_S, hydrogen sulfide; NAC, *N*-acetylcysteine; NAMPT, nicotinamide phosphoribosyltransferase; PAG, propargylglycine; mCAT, mitochondrial catalase; Pitx2, paired-liked homeodomain transcription factor 2; Nrf2, nuclear factor-erythroid-2-related factor 2; CDK, cyclin-dependent kinases; HBP, hexosamine biosynthesis pathway; Pck2, phosphoenolpyruvate carboxykinase 2; OGA, *O*-GlcNAcase. The yellow sun represents the promotion of heart regeneration, while the red lightning represents the inhibition of heart regeneration.

**FIGURE 4:**
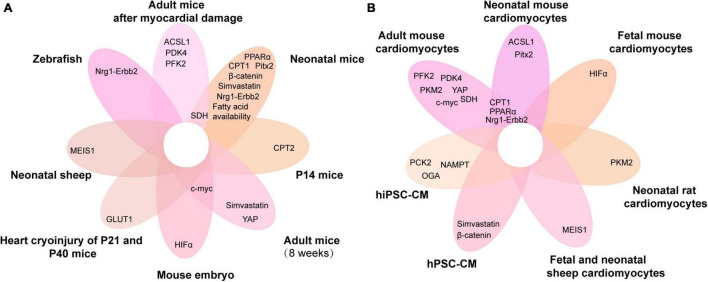
Venn diagram of metabolic genes associated with cardiomyocyte proliferation overlapping in different animals and cardiomyocytes. **(A,B)** The metabolic genes associated with cardiomyocyte proliferation in different animals **(A)** and cardiomyocytes **(B)**. ACSL1, acyl-CoA synthetase long-chain family member 1; PFK, phosphofructokinase 2; PPARα, peroxisome proliferator-activated receptor; HIF-α, hypoxia-inducible factor α; PDK4, pyruvate dehydrogenase kinase 4; MEIS1, myeloid ecotropic viral integration site 1; PCK2, phosphoenolpyruvate carboxykinase 2; CPT, carnitine palmitoyltransferase; NAMPT, nicotinamide phosphoribosyltransferase; YAP, yes-associated protein; Nrg1, neuregulin 1; SDH, succinate dehydrogenase; GLUT1, glucose transporter 1; Pitx2, paired-like homeodomain 2; hiPSC-CM, human-induced pluripotent stem cell cardiomyocytes; hPSC-CM, human pluripotent stem cell-derived cardiomyocytes; P14, postnatal day 14; P21, postnatal day 21; P40, postnatal day 40.

Heart failure is by far the leading cause of mortality and disability throughout the world. Neonatal mice have an ability of myocardial regeneration and thus serve as a model for the treatment of heart failure. In this review, we summarize the current status of research on the metabolic regulation of myocardial regeneration, including recent findings and controversies (Table 1 and Figure 3). Glycolipid–amino acid metabolism and biosynthesis are critical for the regulation of heart regeneration. Based on this, changing metabolism of related components, making cardiomyocytes to re-enter the cell cycle, promoting proliferation of cardiomyocytes, and enhancing cardiac regeneration serve as effectives therapies for heart diseases that eventually evolve into heart failure (Figure 4 and Table 1). However, further studies are required to find solutions for the following problems: For example, several metabolic pathways are closely related to each other, so which molecule or mechanism controls the direction of metabolic changes? What are the mechanisms by which the metabolic alterations eventually affect the cardiomyocytes proliferation and heart regeneration? Many metabolisms end up affecting the intra-nuclear epigenetic mechanisms. So, whether the combination of metabolism and epigenesis influences the process of heart regeneration? In addition to the four major metabolisms discussed in this review, are other metabolic pathways involved in the regulation of cardiac regeneration? For example, is the current and popularly studied novel metabolism of metal ions (iron and copper metabolism) related to other heart diseases?

**TABLE 1:** Summary of recent studies demonstrating the role of metabolism in heart regeneration.

Metabolic pathway	Species	Cells	Intervention targets	Results	References
Fatty acid oxidation	Postnatal mice	—	Fatty acid availability	Fatty acid-deficient milk prolonged the proliferation window of their hearts	(63)
	Infant mice	Primary neonatal mouse cardiomyocytes and primary 3-week-old mouse cardiomyocytes	Carnitine palmitoyltransferase 1 (CPT1)	Inhibition of the activity of CPT1 reduced fatty acid oxidation and increased cardiomyocyte proliferation	(61)
	P14 mice	—	Carnitine palmitoyltransferase 2 (CPT2)	Partial depletion of CPT2 enhanced cardiomyocyte proliferation	(66)
	Adult mice post-MI	Primary neonatal mouse cardiomyocytes	Acyl-CoA synthetase long-chain family member 1 (ACSL1)	ACSL1 knockdown inhibited fatty acid oxidation and enhanced the cardiomyocyte proliferation	(67)
	Infant mice	Primary neonatal mouse cardiomyocytes and primary 3-week-old mouse cardiomyocytes	Peroxisome proliferator-activated receptor (PPAR) α	PPARα-mediated fatty acid β-oxidation promoted proliferation of cardiomyocytes in P4 mice, while it enhanced the presence of binucleated cardiomyocytes in P5 mice	(61)
Glycolysis	Mouse embryo	Fatal mouse cardiomyocytes	Hypoxia-inducible factor α (HIF-α)	HIF-1α deficiency impaired glycolysis in mouse heart and inhibited cardiomyocyte proliferation	(19, 49, 50)
	Heart cryoinjury of P21 and P40 mice	—	Glucose transporter 1 (GLUT1)	The increase in glucose metabolism mediated by Glut1 overexpression promoted cardiac regeneration in neonatal mouse heart	(9)
	Adult mice after myocardial ischemia-reperfusion	Isolated adult mouse cardiomyocytes	Phosphofructokinase (PFK) 2	PFK2 overexpression increased the contractility of hypoxic cardiomyocytes	(74)
	Adult mice post-MI	Primary adult mouse cardiomyocytes	Pyruvate dehydrogenase kinase (PDK) 4	PDK4 deletion decreases DNA damage and promotes cardiac regeneration	(63)
	Zebrafish and adult mice post-MI	Neonatal rat CMs and adult mouse CMs	M2-pyruvate kinase (PKM2)	Pkma2 knockdown in zebrafish decreased glycolysis and inhibited cardiomyocyte proliferation; overexpression of PKM2 promoted cardiomyocyte proliferation and regeneration after MI in adult mice	(68, 78)
	Neonatal sheep	Primary fetal and neonatal sheep cardiomyocytes	Myeloid ecotropic viral integration site 1 (MEIS1)	Inhibiting MEIS1 expression promoted sheep cardiomyocyte maturation by decreasing glycolytic genes expression	(80)
	Adult murine heart	Adult mouse cardiomyocytes	Yes-associated protein (YAP)	Activation of YAP enhanced glycolysis and promoted cardiomyocyte proliferation	(84–86)
	Neonatal mice and zebrafish	Neonatal, juvenile and adult CMs	Neuregulin 1 (Nrg1)- Erbb2	Activation of Nrg1-Erbb2 enhanced glycolysis and promoted cardiomyocyte proliferation	(88, 89)
	Neonatal mice	Immature human pluripotent stem cell-derived cardiomyocyte (hPSC-CM)	β-catenin	Activation of β-catenin enhanced glycolysis and promoted cardiomyocyte proliferation	(91)
	Fatal and adult mice	Islet1^+^ cardiac progenitors and primary adult mouse cardiomyocytes	c-Myc	Activation of c-Myc enhanced glycolysis and promoted cardiomyocyte proliferation	(92)
TCA cycle metabolism	Neonatal mice	Primary neonatal mouse cardiomyocytes	Paired-like homeodomain 2 (Pitx2)	Pitx2 knockdown inhibited myocardial regeneration of neonatal mice through producing ROS	(84)
	Adult mice after MI and neonatal mice	Adult mouse cardiomyocytes	Succinate dehydrogenase (SDH)	Malonate (SDH inhibitor) promoted adult mouse cardiomyocyte proliferation by reducing succinate accumulation	(107)
Anabolic pathways	Neonatal mice and adult mice	Human PSC-cardiac organoids and hPSC-derived cardiomyocytes (hPSC-CM)	Simvastatin	Simvastatin suppressed cardiomyocyte proliferation by inhibiting the mevalonate pathway of isoprene synthesis	(110)
	—	Human iPSC cardiomyocytes (hiPSC-CM)	*O*-GlcNAcase (OGA)	Overexpression of *O*-GlcNAcase (OGA) was associated with HBP and prevented cardiomyocyte cell cycle entry	(111)
	—	Human iPSC cardiomyocytes (hiPSC-CM)	Nicotinamide phosphoribosyltransferase (NAMPT)	Knockdown of (NAMPT) inhibited cardiomyocyte cycle entry	(111)
	—	Human iPSC cardiomyocytes (hiPSC-CM)	Phosphoenolpyruvate carboxykinase 2 (PCK2)	Overexpression of PCK2 promoted proliferation of hiPSC-CM	(111)

The primary step of heart regeneration is to promote the cardiomyocytes to enter the cell cycle for division and proliferation. The interphase of the cell cycle is metabolically active and is the period during which the cells synthesize various enzymes, RNA, DNA, and proteins. During the S-phase, the cell cycle can be promoted by metabolic enzymes and proliferation pathways that target nucleic acid and protein synthesis, thereby promoting entry of cardiomyocytes into the cell cycle. However, specific biosynthetic pathways and metabolic enzymes need to be further explored, in order to increase the proliferative capacity of cardiomyocytes.

Certain questions are still unanswered. For example, are there other cardiac component cells (such as immune cells, endothelial cells, and fibroblasts) that interact with cardiomyocytes to influence their metabolism and thus alter their proliferative potential? Macrophages are required for myocardial regeneration in neonatal mice (137). However, it is unclear whether macrophages reprogram cardiomyocyte metabolism through intercellular interaction or paracrine effects. The angiogenesis of vascular endothelium supports regenerating cardiomyocytes after MI (138). The vascular endothelium supplies paracrine factors to cardiomyocytes so that they participate in regeneration. However, whether it affects the metabolism of cardiomyocytes is unclear. Besides, the investigators suggest that *in situ* modulation of cardiac cells in infarct foci can promote cardiomyocyte proliferation to repair scars and thus avoids the need of transplantation (139). Cardiac fibroblasts constitute a large proportion of the cardiac cells, and previous studies suggest that the cocktail therapy could potentially reprogram the fibroblasts in infarct foci directly into cardiomyocytes by modulating the transcription factors (139). Nonetheless, it is unclear whether cardiomyocyte metabolism is altered in this case? In addition, is it possible to enhance the proliferation of cardiomyocytes directly with small molecule drug-targeted metabolites?

Currently, hypoxia-induced glycolysis is the most studied among the metabolic pathways and has the greatest impact on myocardial regeneration. In future, translational studies can be conducted in depth from glycolysis to provide new targets for future clinical translation. It is important to further explore the relationship between myocardial regeneration mechanisms and changes in myocardial energy metabolism. However, we should note that almost none of the published studies have proven that their manipulations improve cardiac function by directly increasing the number of cardiomyocytes. The improved heart function is mainly correlated with the enhanced cell cycle activity and the improved functioning of the remaining cardiomyocytes, which might be due to a metabolic shift. Thus, it is essential to uncover the signaling pathways and key regulatory factors that affect energy metabolism accompanied by myocardial regeneration to develop effective therapeutic approaches. In all, we are optimistic that it will be possible to achieve great progress in human heart regeneration.

## Author Contributions

ZZ and XL conceived and designed the study and revised the manuscript. XD analyzed the data and wrote the manuscript. All authors listed and approved the manuscript for publication.

## Conflict of Interest

The authors declare that the research was conducted in the absence of any commercial or financial relationships that could be construed as a potential conflict of interest.

## Publisher’s Note

All claims expressed in this article are solely those of the authors and do not necessarily represent those of their affiliated organizations, or those of the publisher, the editors and the reviewers. Any product that may be evaluated in this article, or claim that may be made by its manufacturer, is not guaranteed or endorsed by the publisher.
